# RNA-binding proteins in mouse male germline stem cells: a mammalian perspective

**DOI:** 10.1186/s13619-015-0022-y

**Published:** 2016-02-02

**Authors:** Huayu Qi

**Affiliations:** Key Laboratory of Regenerative Biology, Guangdong Provincial Key Laboratory of Stem Cell and Regenerative Medicine, Guangzhou Institutes of Biomedicine and Health, Chinese Academy of Sciences, Guangzhou, 510530 China

**Keywords:** Adult stem cells, RNA-binding proteins, Post-transcriptional regulation, Translational regulation, Protein synthesis

## Abstract

Adult stem cells that reside in particular types of tissues are responsible for tissue homeostasis and regeneration. Cellular functions of adult stem cells are intricately related to the gene expression programs in those cells. Past research has demonstrated that regulation of gene expression at the transcriptional level can decisively alter cell fate of stem cells. However, cellular contents of mRNAs are sometimes not equivalent to proteins, the functional units of cells. It is increasingly realized that post-transcriptional and translational regulation of gene expression are also fundamental for stem cell functions. Compared to differentiated somatic cells, effects on cellular status manifested by varied expression of RNA-binding proteins and global protein synthesis have been demonstrated in several stem cell systems. Through the cooperation of both cis-elements of mRNAs and trans-acting RNA-binding proteins that are intimately associated with them, regulation of localization, stability, and translational status of mRNAs directly influences the self-renewal and differentiation of stem cells. Previous studies have uncovered some of the molecular mechanisms that underlie the functions of RNA-binding proteins in stem cells in invertebrate species. However, their roles in adult stem cells in mammals are just beginning to be unveiled. This review highlights some of the RNA-binding proteins that play important functions during the maintenance and differentiation of mouse male germline stem cells, the adult stem cells in the male reproductive organ.

## Introduction

**Fig. 1:**
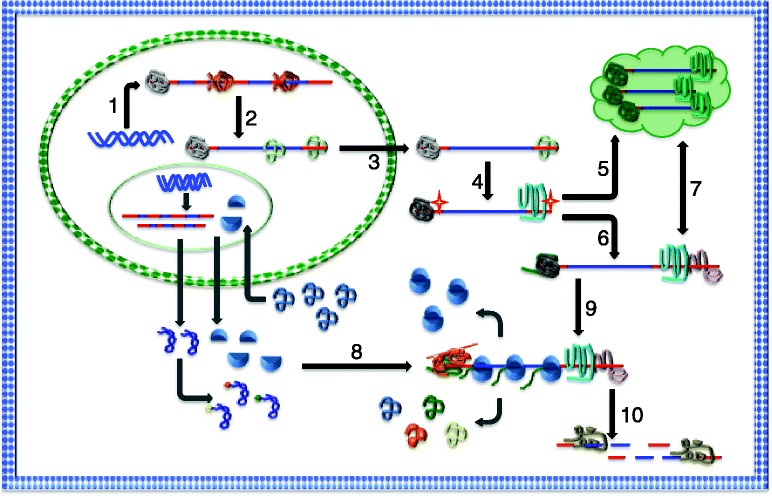
The life cycle of mRNAs. mRNAs undergo a series of modification events since they are transcribed from the genome. These processes are facilitated by the action of numerous RNA-binding proteins (RBPs) (shown as *molten globules* in the diagram), which interact with mRNAs at various regions through conserved RNA-binding domains. Interactions with RBPs and associated proteins render status of mRNAs as either repressive or active for protein synthesis in the cytoplasm of a cell. mRNAs can be stored in large RNA-protein complexes (RNA granules, *cloud in green*) in the cytoplasm when translation is not permitted. The dynamic exchange of mRNAs between cytoplasm and RNA granules is mediated by RBPs that are not fully characterized. Translational machinery, including tRNAs, ribosomal RNAs, and subunits are synthesized in the nucleolus and exported to cytoplasm in order for protein synthesis to occur. Following translation, tRNAs and ribosomal subunits can be recycled for additional rounds of translation. Major processes of mRNAs’ life cycle are indicated in *numbers* (*black arrows*). (*1*) Transcription; (*2*) splicing; (*3*) nuclear export; (*4*) post-transcriptional modification of mRNAs; (*5*) cytoplasmic ribonucleoprotein complex (RNA granule) formation; (*6*) cytoplasmic alternative polyadenylation (APA); (*7*) exchange of mRNAs between RNA granule and cytoplasm; (*8*) complex formation at the 5′- and 3′-UTRs of mRNAs, translation initiation; (*9*) translation; and (*10*) degradation. *Blue rod*: exons; *red rod*: untranslated regions of mRNA

Tissue homeostasis and regeneration require balanced regulation of self-renewal and differentiation of adult stem cells (ASCs) that reside in particular tissues. Like any other cell type, cellular functions of ASCs depend on specific gene expression programs that are subject to precise control in order to produce necessary and sufficient proteins, the functional units within cells. In response to environmental stimuli, genetic information is transferred to proteins through messenger RNA (mRNA) production (transcription) and protein synthesis (translation) under the regulation of cell-autonomous and non-autonomous factors in order for cells to elicit proper functions. Intensive investigations over past decades have uncovered key factors and molecular mechanisms that govern the regulation of gene expression during self-renewal and differentiation of ASCs, of which transcription factors and transcriptional regulation have been at the center stage. However, abundance of mRNAs in a cell is not necessarily equivalent to the abundance of functional proteins that cells produce. From the birth of mRNAs to their translation and eventual degradation, mRNAs undergo extensive modifications and regulation, mainly through the action of RNA-binding proteins (RBPs) (Fig. 1). Since cells often dedicate ~20 % of their cellular energy to the process of protein synthesis, regulation of gene expression at the post-transcriptional and translational levels are thus of great importance. It has been increasingly realized that post-transcriptional and translational regulation hold fundamental roles in stem cells [1, 2]. Global effects of protein synthesis on stem cell behavior manifested by RBPs and translational regulation have been demonstrated in several stem cell systems [3–5]. However, how RBPs participate in various steps of RNA metabolism during self-renewal and differentiation of ASCs and how ASCs are regulated at the post-transcriptional and translational levels in order to accommodate tissue homeostasis and regeneration remain largely unexplored.

**Fig. 2:**
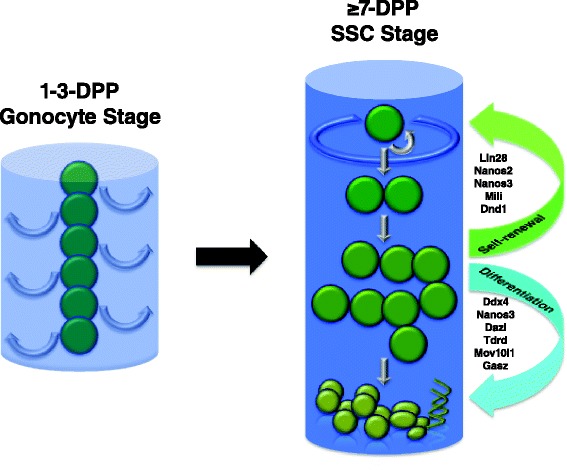
Mouse spermatogonial stem cells. Gonocytes (descendents of PGC in male embryonic gonad, also called prospermatonia) resume mitotic cell division and migrate from the center of growing seminiferous tubule to the basement membrane during the first 3 days following the birth of the animal. Spermatogonial stem cell (SSC) colonies are established around 7 dpp (days post-partum) at the inner surface of seminiferous tubule. They both undergo self-renewal to replenish stem cell pool and differentiation toward the lumen in order to generate sperm cells throughout the life of adult animal. RNA-binding proteins (depicted in the diagram) participate in the regulation of self-renewal and differentiation of SSCs

Germline stem cells in adult animals are ASCs in reproductive organs and have been one of the widely utilized systems for stem cell research. In mouse embryos, primordial germ cells (PGCs) are formed around E6.25 from proximal posterior epiblast. They then proliferate and migrate into embryonic gonad to form either prospermatogonia or oogonia in male and female animals, respectively. In males, prospermatogonia (also called gonocytes) are the precursor of future spermatogonial stem cells (SSCs) in adult animals. Quiescent gonocytes in the embryo (arrested at prophase of mitotic cell cycle) only resume cell division following birth of the animal. During the first 3 days of post-natal development (1–3 dpp (days post-partum)), gonocytes proliferate and migrate from the center of developing seminiferous tubule to the basement membrane. Colonies of SSCs composed of type A undifferentiated stem cell populations are established around 7 dpp. These cells exist as single cells (A_single_ or A_s_) or cohorts (A_paired_ or A_pr_ and A_aligned_ or A_align_, due to incomplete cytokinesis). Although poorly defined, niche environment consisting of surrounding somatic Sertoli cells, Leydig cells and interstitial Myoid cells provide essential stimuli, such as hormones and growth factors, to regulate the self-renewal and differentiation of SSCs. Previous studies have shown that PGCs, gonocytes, and SSCs all possess characteristics of stem cells, although with varied degree of pluriopotency, based on examinations of their differential gene expression and in vitro tests. Nevertheless, SSCs undergo self-renewal and differentiation and are the bases for continuous production of spermatozoa (matured sperm) throughout animal’s adult life (Fig. 2).

It has been shown that RBPs play pivotal functions during germ cell development. Their participation in the regulation of self-renewal and differentiation of germline stem cells are first demonstrated in invertebrates, such as *Drosophila* and *Caenorhabditis elegans* [5, 6]. Relatively less is known about functions of RBPs in germline stem cells in mammals. Increasing evidences show that mammalian germ cells regulate their overall development utilizing not only general machineries for RNA metabolism and translation but also germline specific mechanisms. Small non-coding RNAs, such as miRNAs and piRNAs, are particularly enriched in spermatogenic cells. Disruption of small RNA synthesis showed deleterious effects on spermatogenesis in mouse [7–9]. Recent studies further showed that long non-coding RNAs (lncRNAs, >200 bps) participate in various steps of spermatogenesis. Some of the newly identified lncRNAs are specifically expressed in germ cells. Current advances on this frontier have been summarized in a recent review [10]. In female germline, post-transcriptional regulations have been shown to be essential for female germ cell development. Some of the RBPs that function in female germline were also found to be important for the male counterpart, while others were specific to female germ cells [11].

In male germline stem cells, RBPs have been shown to participate in various processes throughout the life cycle of mRNAs during mammalian germ cell development, ranging from transcription (such as DDX21) to translational activation (such as LIN28). They interact with non-coding RNAs or mRNAs in order to modulate the stability of RNA species (by forming ribonucleoprotein complexes, RNPs), repress transposable elements (TEs) in germline to protect genome integrity, and direct protein translation in a spatial-temporal manner. In this review, known RBPs that have been shown to directly influence the maintenance and differentiation of spermatogonial stem cells in mouse are highlighted. Studies of these RBPs demonstrate some common molecular mechanisms by which they function. Combining this current knowledge and the latest development of research technologies, exciting opportunities present in front of us to further elucidate unknown players and their functions.

### RNA-binding proteins in mouse male germline stem cells

**Fig. 3:**
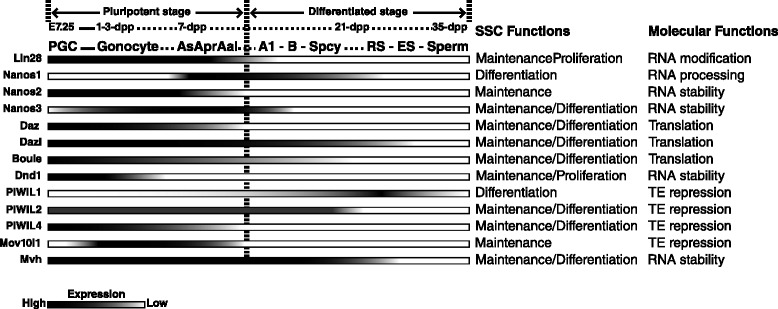
RBPs in mouse male germline. Diagram of temporal expression patterns of known RNA-binding proteins and their functions during mouse spermatogenesis. Developmental times and various types of male germline cells are indicated above the expression patterns of RBPs (*graded bars*) during germ cell development. Functional involvement of the RBPs during maintenance and differentiation of spermatogonial stem cells (SSCs) and RNA metabolism are summarized in the *middle* and *right panels*, respectively. *PGC* primordial germ cells, A_s_, A_pr_, A_al_: undifferentiated spermatogonial stem cells; A1, B: type A1 and type B differentiating spermatogonia; *Spcy* spermatocyte, *RS* round spermatid, *ES* elongating spermatid (different from the embryonic stem cells in the text), *TE* transposable element

“Inert genome” theory was put forth in 1980s to explain the differences between cell fate determination of germline cells and somatic cells [12, 13]. It suggested that genome of germline cells are “inert” and thus hard to change or express, while somatic cells contain genomes that are modified toward different cell states. This allows germline cells to retain higher developmental potency, comparable to that of embryonic stem cells, and also illustrates the importance of regulatory mechanisms outside of genome in germ cells. Research in the past decades demonstrated critical functions of several RBPs during maintenance, proliferation, survival, and differentiation of germline stem cells. Their temporal expression patterns are well-coincided with their functional involvement during spermatogenesis (Fig. 3).

#### LIN28

LIN28 protein has two isoforms, LIN28A and LIN28B. LIN28A contains CCHC-type zinc finger RNA-binding domain and expressed primarily in germline. Its functional role as a pluripotent factor has been widely recognized. Yu et al. used LIN28A, in combination with OCT4, SOX2, and NANOG, to successfully convert human fibroblasts into pluripotent stem cells (hiPSCs) [14]. It was found that, in embryonic stem cells (ESCs), LIN28A binds the 5′-GGAGA-3′ at the 3′-terminal loop of pre-let-7 miRNA precursor and recruits TUTase4 3′-terminal uridylyl transferase (zinc finger, CCHC domain containing 11 (ZCCHC11)) that uridylates pre-let-7 [15]. This prevents further processing of the miRNA precursor by Dicer and eventual degradation of uridylated pre-let-7. Via binding of the same 3′-loop region, TRIM25, an E3 ubiquitin ligase turned RNA-binding protein, confers LIN28A/TUTase4 uridylation activity specifically to pre-let-7 [16], providing additional layer of specificity control. In addition to its miRNA-binding activity, LIN28A interacts directly with *Pou5f1* mRNA in the CDS. Together with RNA helicase A (RHA), this interaction with the mRNA promotes protein translation of *Pou5f1* [17]. Thus, LIN28A can regulate the self-renewal and maintenance of stem cells via inhibition of let-7 production and activation of pluripotent gene expression. In neural stem cells, miR125 could target *Lin28a* mRNA and thus relieve the stress on let-7. This forms a negative feedback loop that regulates the self-renewal and differentiation of neural stem cells [18]. Functional screens using siRNAs showed that LIN28A affects PGC development in vitro [19]. It was found that LIN28A could enhance the conversion of PGC from embryonic stem cells. Its role in germline pluripotency and animal development in vivo are further emphasized by genetics studies in mouse. Deletion of *Lin28a* gave rise to mutant mice containing reduced PGC population and fertility. When conception succeeded, animals die at post-natal age due to massive metabolic and growth defects [20].

As an RNA-binding protein, the roles of LIN28A extend beyond stem cells. Since the discovery of its role during cell growth and differentiation in *C. elegans*, LIN28 has been shown to modulate cellular metabolism and growth in somatic and pluripotent stem cells through its targeting of Insulin/PI3K/mTOR pathway components and metabolic enzymes [21]. Combined RNA sequencing and bioinformatics analyses showed that LIN28A interacts directly with RNA species that contain conserved sequence motif 5′-GGAGA-3′ or LRE (LIN28-responsive element). These studies identified a variety of mRNAs as LIN28A targets, including mRNAs for translation regulators, splicing factors, and cell cycle controllers [22–24]. Its role, however unclear, is to stabilize mRNAs and enhance protein translation, possibly at the initiation stage. In consistent with this notion, LIN28A has been found to co-sediment with polyribosomes on sucrose gradient of cell lysates [22]. Interestingly, LIN28A also functions on the other side of the coin. It was found that LIN28A suppresses translation of proteins that are associated with ER, Golgi, and secretory pathways in embryonic stem cells via binding of non-canonical sequences within terminal loop of a small hairpin of target mRNAs [25]. The less recognized LIN28B contains cold shock RNA recognition domain and has been implicated in tumor progression process as an oncogene. It appears that Lin28 proteins elicit their functions in a target-specific and cell context-dependent manner. How LIN28 is regulated for its versatile functions remains an intriguing question. Perhaps more stringent conditions, such as conditional knockout mouse, will be helpful in elucidating the mechanisms underlying LIN28 functions and its role in germline stem cells. Additional co-factors and post-translational modifications of the proteins themselves may be part of the functional system of LIN28.

#### NANOS family

NANOS proteins are zinc finger containing RNA-binding proteins, specifically expressed in germline. It contains three proteins (NANOS1, NANOS2, and NANOS3) that are important for the maintenance of male germline stem cells and spermatogenesis. Cell lineage tracing suggested that NANOS2 and NANOS3 are expressed in undifferentiated SSCs at different stages. While NANOS2 is mainly expressed in A_s_ and A_pr_ SSCs, NANOS3 is expressed in all undifferentiated SSCs, suggesting their common and yet different roles in regulating SSCs [26]. Functions of NANOS2 were elucidated in studies using mutant and transgenic mice. In *Nanos2* mutant mice, germ cells developed pre-maturally at pre-natal stage with meiosis commenced in gonocytes. On the other hand, selective deletion of *Nanos2* in mouse testis caused accumulation of differentiating SSCs and gradual loss of spermatogenesis [27]. In contrast, over-expression of *Nanos2* caused accumulation of PLZF^+^ SSCs (mainly A_s_ and A_pr_) in seminiferous tubules without increased cell proliferation or decreased apoptosis. This subsequently caused reduced germ cell differentiation. In addition, Sada et al. found that over-expression of *Nanos2* partially rescued GFRα1^−/−^ phenotype, placing NANOS2 downstream of GDNF in the SSC self-renewal pathway [28]. These observations suggested that NANOS2 plays important functions in repressing meiotic gene expression and SSC maintenance. Deletion of *Nanos3* in mouse caused increased apoptosis in PGC that is under the control of both BAX-dependent and independent pathways [29].

Both NANOS2 and NANOS3 are found in ribonucleoprotein complex (RNA granules) in the cytoplasm of embryonic and neonatal male germ cells. However, these RNA granules differ from the mouse VASA homologue (MVH)-containing chromatoid body (CB, a large RNP complex in haploid spermatids) suggesting that they may regulate different sets of mRNAs in germline stem cells. Biochemical analyses using mass spectrometry following protein co-immunoprecipitation identified proteins that interact with NANOS2 [30]. Among them, CCR4-NOT deadenylase complex co-localize with NANOS2 in P-body of spermatogenic cells. P-body in *Nanos2* mutant mice appeared aberrant in shape and number without CCR4-NOT, indicating that NANOS2 not only maintains P-body integrity but also facilitates the localization of CCR4-NOT complex. This P-body effect is directly associated with the RNA-binding activity of NANOS2 since zinc finger truncation mutation of NANOS2 showed the similar phenotype [31]. In addition, GST pull-down assays also suggested that NANOS3 could interact with CNOT8 [31]. Because the CCR4-NOT complex contains RNA deadenylase activity, it is proposed that NANOS2 and NANOS3 mediate the degradation of target mRNAs via interactions with CCR4-NOT. Indeed, it was found that mRNAs of meiotic genes were increased in *Nanos2* mutant mice [27]. In the same vein, a recent study showed that NANOS2 was able to retain mTOR in the stress granules of SSCs along with differentiation-related transcripts and thus preventing the translational signaling from activating [32].

However, NANOS2 was also found in polyribosomal fraction of cell lysates, indicating its function in active protein translation [33]. How does NANOS2 affect mRNA dynamics between repressed and activated states remain elusive. Microarray analyses following NANOS2 immunoprecipitation revealed that its target mRNAs include mRNAs for pluripotent gene *Sox2*, as well as meiotic genes *Stra8* and *Taf7l* [34]. This suggested that perhaps NANOS2 functions as the keeper of tissue specific stem cells. It maintains pluripotency of SSCs while keeping the meiotic potential of germ cells in check. How the repressive and active roles of NANOS2 on protein translation are regulated during maintenance and differentiation of germline stem cells require further investigation.

Less is known about NANOS1 during germ cell development. Preliminary studies suggested that NANOS1 could interact with DEAD-box RNA helicase GEMIN3 and co-localize in the CB of human spermatids, suggesting its role in mRNA processing during the late phase of spermatogenesis [35]. Human syndrome of azoospermia and oligozoospermia has recently been linked to mutations that occur in *Nanos1* [36]. It is not clear whether NANOS1 also binds CCR4-NOT complex to mediate degradation of target mRNAs.

#### DAZ family

Deleted in azoospermia (DAZ) family proteins contain three members (DAZL, DAZ, and BOULE) that interact with ribonucleic acids via the conserved RNA recognition motif. They share high degree of homology (up to 80 %) among themselves. DAZ proteins are specifically expressed in germline of both sexes from embryonic till post-meiotic stage and play important functions in regulating germ cell development. As RNA-binding proteins, they elicit their roles on germ cell development through modulating the translation of specific mRNAs. Using microarray analyses following protein immunoprecipitation, it was found that DAZL regulates the translation of germ cell-specific genes, such as *Mvh*, through direct interaction with their mRNAs [37]. In human ESCs cultured in vitro, over-expression of DAZ family proteins induced haploid cell formation. Concomitant with DAZL expression in hESCs, VASA expression was also induced, while knockdown of *Dazl* reduced VASA by ~50 % [38]. In addition, expression of pluripotent genes that are normally expressed in germline stem cells were also induced with the expression of DAZ proteins in ESCs, consistent with the notion that DAZ proteins regulate germ cell fate determination. Like NANOS and LIN28, DAZL activates translation of mRNAs depending on cellular context and its targets. It was found that DAZL binds 3′-UTR of germ cell mRNAs and PABP during translation initiation [39, 40]. These studies suggested that DAZL interacts with consensus motifs within 3′-UTR of target mRNAs and facilitate their protein expression probably via interactions with initiation complex. It is not clear whether there are other consensus RNA sequences for DAZL interaction and whether they are conserved in other target mRNAs.

DAZL was found to localize in stress granule, the RNA-protein complex that forms in cells’ cytoplasm when under stress. It helps to maintain mRNAs that are not actively translated or when repression is required. In *Dazl* mutants, stress granules were diminished and mRNAs and translation inhibitor phosphorylated eIF2α could not be recruited to stress granules [41]. The stress granule localization of DAZL suggests its repressive role in mRNA translation. This is in consistence with the observation that DAZL targets several pluripotent genes (including *Sox2* and *Sall4*, but not *Pou5f1*) and represses their expression at the post-transcriptional level [42]. Thus, DAZL can also function as both positive and negative regulators of mRNA translation. However, it is not clear whether DAZL is also present in active polyribosomal complexes in cells. Less is known about the other two members of DAZ family, although data suggested that they both are required for germ cell development. For example, human DAZ1 is specifically expressed in pre-meiotic spermatogonia [43]; deletion of *Boll* (*Boule* homologue in *Drosophila*) caused defects in G2/M transition of meiosis in germ cells [44]. Molecular mechanisms of DAZ protein functions require further exploration.

#### DND1

Dead end homologue 1 (DND1) protein is a germ cell-specific RNA-binding protein, containing conserved RNA recognition motif. First cloned from Ter mutant mouse, it was found that a point mutation occurring in *Dnd1* gene generates an early termination codon and is responsible for the testicular germ cell tumor (TGCT) phenotype resembling that of Ter mutation in human [45]. TGCT is caused by aberrant germline stem cell functions during animal’s fetal development. Over-proliferated germline stem cells migrate and integrate into various types of tissues and cell lineages, leading to multiple tumor growth. In *Ter*/*Ter* mutant mice, PGCs were either lost or become embryonic carcinoma integrated into multiple tissues and cell types, similar to the syndrome of human disease. This provided an opportunity to identify the molecular mechanisms that cause the disease. It has been shown that DND1 could bind uridine-rich region of 3′-UTR of mRNAs and prevents miRNA mediated mRNA decay, including *Nanos1* in zebrafish embryo and several pluripotent mRNAs in porcine oocytes [46]. While its in vivo target mRNAs remain to be characterized, DND1 was found to express in ESCs and interact with mRNAs of pluripotent genes (including *Pou5f1*, *Sox2*, and *Nanog*), cell cycle regulators and genes involved in regulating apoptosis in stably transfected ES cells [47]. DND1 itself can be regulated by miRNA and its activity by competing enzyme APOBEC3 that binds the same uridine-rich 3′-UTR region [48, 49]. Thus, DND1 could protect mRNA targets against certain miRNA species in a context-specific manner through direct interaction with mRNAs that affecting proliferation and maintenance of germline stem cells.

#### PIWI family

PIWI/argonaute family proteins are widely recognized for their roles in regulating stem cells and germ cell development. PIWI proteins belong to the subclass of PIWI/argonaute family that interacts with small non-coding RNAs and expresses mainly in the germline. Mouse PIWI family is composed of three proteins, namely PIWI-like protein-1 (PIWIL1, also known as MIWI), PIWI-like protein-2 (PIWIL2, also known as MILI), and PIWI-like protein-4 (PIWIL4, also known as MIWI2), all of which contain conserved PAZ and PIWI domains. Their primary functions in the germline have been repression of TEs on the post-transcriptional level and preventing TEs from sabotaging genomic integrity via binding to PIWI-interacting non-coding RNAs (piRNAs, 26–30 nt long). Recent research extended functions of PIWI proteins to directly regulate mRNA metabolism and epigenetic modifications of the genome. This is mainly achieved through the recognition of piRNA complementary sequences within mRNA and DNA, in collaboration with PIWI-interacting proteins, including RNA helicases and methylation enzymes. More details can be found in a recent review [50].

In mouse, PIWIL1 is specifically expressed in meiotic spermatogenic cells and post-meiotic spermatids. The development of haploid spermatids is halted at early phase of growth in the absence of PIWIL1, suggesting its role in regulating post-meiotic development of sperm [51]. Its localization in the CB implicates its function in maintaining mRNA stability and translational repression, in addition to TE regulation. Interestingly, it was found that PIWIL1 co-sediments with polyribosomes on sucrose gradient of testis lysates and presents in the mRNA cap-binding protein complexes, suggesting its participation in active translation [52]. Experiments in author’s lab also suggested that PIWIL1 interacts with PABPC1 during spermiogenesis [53]. How their interactions affect protein translation during spermiogenesis requires further exploration.

The functional importance of PIWI proteins in germline stem cells has also been elucidated for the other two family members, PIWIL2 and PIWIL4. Both are expressed in germline in PGCs at embryonic stage and meiotic germ cells in adult animals. Results showed that they not only utilize piRNAs as functional accessories but also actively participate in the biogenesis of piRNAs [54, 55]. In consistence with this, germ cells in both *Piwil2* and *Piwil4* mutant mice contained increased level of TEs and decreased production of piRNA species [56, 57]. In either *Piwil2* or *Piwil4* mutant mice, spermatogenesis is disrupted at early prophase of meiotic division, but no apparent defects were found in either PGCs or female germ cells [58, 59]. Aberrant expression of meiotic genes and cellular apoptosis eventually lead to progressive loss of male germ cells [59, 60]. In *Piwil2* mutant, majority of spermatogonia underwent slower cell cycle progression while the perinatal development of gonocytes seemed normal, suggesting that PIWIL2 is important for the maintenance and differentiation of SSCs. The compromised defects in SSCs of these mutant mice suggest that PIWIL2 and PIWIL4 may have different and yet redundant functions in germline stem cells, which could be revealed by studying the mice lacking both genes. Similar to *Mvh* knockout mice, expression of pluripotent gene *Pou5f1* was decreased in *Piwil2* mutants (but increased in *Piwil4* mutant), consistent with its role in regulating stem cells [54, 60]. Both PIWIL2 and PIWIL4 were found in the CB, where they interact with MVH and Tudor proteins [54]. Intriguingly, PIWIL2 was found to interact with protein translation regulators, including eIF3A, eIF4E, eIF4G, and m7G-cap complex in an RNA-dependent manner [60]. The overall protein synthesis was reduced in *Piwil2* mutant, supporting its role in translational regulation. What molecular mechanisms are underlying translational control by PIWI proteins and how these affect germline stem cells are fascinating questions for future research.

#### Other transposable element regulators

Moloney leukemia-activated virus 10-like 1 mouse homologue (MOV10L1) is a testis- and heart-expressing ATP-dependent DExD box RNA helicase. Although functions of MOV10L1 are not completely understood, it has been shown that MOV10L1 interacts with MILI and MIWI to facilitate piRNA biogenesis, thus may be important for the TE control and genome maintenance in male germ cells [61]. Gene deletion in mouse caused aberrant expression of Line1 and IAP, two transposable elements in the male germline. These mice contain germ cells that are arrested at early meiotic stage [62, 63]. Genetic studies further showed that RNA helicase domain of MOV10L1 is important for its piRNA processing activity and thus germ cell development [61, 63]. In addition, it was found that MOV10L1 co-localizes with germ cell protein with ankyrin repeats, sterile-α motif, and leucine zipper (GASZ) (see below) in the cytoplasm, implicating its role in mitochondrial regulation.

GASZ was first found in 2002 during screening of genes that are specifically expressed in germ cells. The protein is highly conserved in different species, ranging from *Xenopus* to human. Although its function is not fully understood, germ cells of *Gasz* mutant mice were arrested at early meiotic stage and contained increased levels of transposable elements and decreased expression of piRNAs and nuage proteins [64], suggesting its participation in the TE regulatory pathway. It was found that GASZ associates with MILI containing RNA granules and partially overlaps with MVH in spermatocytes. However, it was not located in the CB. In the absence of *Gasz*, both MILI and MVH were greatly reduced in RNA granules, suggesting that GASZ may participate in the organization of RNA granules in meiotic germ cells. Recent results suggested that GASZ may bind DAZL and facilitate the germ cell fate determination and expression of pluripotent genes in embryoid bodies cultured in vitro [65]. Can it maintain germline stem cells via regulating pluripotent gene expression and meiotic genes like NANOS and DAZL? Further research will provide the answers.

#### MVH

MVH, also known as DDX4, interacts with ribonucleic acids through conserved RNA-binding motif DEAD (Asp-Glu-Ala-Asp) box. It is an ATP-dependent RNA helicase, which often modifies the secondary structures of RNA during processes such as alternative splicing and protein translation initiation. MVH expression starts in the embryonic germline in PGCs and lasts till the completion of meiosis in male germ cells. It was found that MVH interacts with other RBPs, such as Tudor and PIWI proteins, and co-localizes with them in RNP complexes including the CB. Deletion of *Mvh* gene in mouse interrupted spermatogenesis. Male germ cells stopped to develop beyond zygotene stage of meiosis, causing absence of mature sperm and eventually leading to male sterile phenotype [66]. In these mice, mRNAs of several genes including *Sycp1*, *Sycp3*, *a-myb*, *Hox1.4*, and *Cyca1* were reduced, all of which are important for normal progression of meiosis in germ cells. In addition, male germ cells in mutant mice contain disrupted nuage and CB. These results indicate the importance of MVH in the maintenance of mRNA stability, translation, and TE repression, as well as its role in regulating the RNP structures [67].

Interestingly, PGCs of *Mvh* mutant mice reduced proliferation more than twofold comparing to the wild type during an in vitro proliferation assay. Expression of pluripotent gene *Pou5f1* was also reduced dramatically on E12.5, although AP staining remained the same as in wild type [66]. These suggested that MVH may regulate the proliferation and pluripotency of PGCs. Its role in pluripotency regulation is exemplified in *Planaria*, where increase of MVH was observed during tail regeneration assay, indicating its requirement for proliferation and maintenance of neoblasts, the stem cells in the animal [68]. It is not clear whether MVH directly regulates SSCs, but its effects on mRNAs of meiotic genes may imply that it, at least in part, prevents the differentiation of pluripotent germ cells via selectively regulating the synthesis of proteins that “for” stemness and “against” meiotic differentiation. Microarray analyses following protein immunoprecipitation suggested that MVH targets over 800 mRNA species, including mRNAs for proteins involved in spermatogenesis, energy metabolism and translation regulation [69]. However, even among the mRNAs expressed during meiotic development, MVH selectively targets a subset of them. How is the selectivity of MVH achieved? Studies suggested that functions of MVH may be regulated by post-translational modification of the protein. Both acetyltransferase HAT1 and co-factor P46 modify residue Lys-405 of MVH near the RNA-binding domains and inhibits its RNA-binding activity when necessary [69]. The molecular mechanisms that govern integration of MVH functions during RNA metabolism at varied steps of germ cell development, particularly at the pluripotent stage, remains to be further studied.

#### DDX RNA helicases and Tudor domain proteins

RNA helicases modulate the architecture of RNAs and thus the accessibility of RNAs to proteins, such as RNA modifying enzymes. Meantime, they also directly interact with proteins and bring them to RNA regions that are under regulation. In germ cells, RNA helicases have been found to play pivotal functions during growth and differentiation of germ cells. In human, there are more than 90 DDX helicase genes, of which two thirds are RNA-related [70]. DDX family proteins contain conserved DExH or DExD box for their interaction with RNA, through which they modulate structures of nucleic acids and alter gene expression and protein translation. They often depend on ATP for their helicase activity. The most known DDX RNA helicase in the germline is the aforementioned MVH. DDX proteins affect mRNA metabolism on multiple levels due to their functional diversity. Studies have shown that they could participate in regulating gene expression via direct binding with both DNA and RNA. For example, DDX21 was recently shown to regulate gene expression of ribosomal genes at both transcriptional and post-transcriptional levels [71]. Several DDX proteins were found to interact with miRNAs and mRNAs directly. Through recruiting modulator protein complexes to the 5′- and 3′-UTRs of mRNAs, they regulate mRNA stability and translation efficiency [72, 73]. DDX25 (GRTH) was found to specifically express in spermatocytes and haploid spermatids in mouse. Deletion of the gene led to developmental arrest of early elongating spermatids. Analyses found that in the germ cells of mutant mice, mRNA levels of several meiotic genes were comparable to those of wild type mice, while their respective proteins were depleted, consistent with its localization in the CB and its role in regulating protein translation and germ cell survival [74]. Although no DDX proteins have been shown to directly participate in stem cell regulation in mammals, planarian DDX proteins MVH and Spoltod were found to be important for self-renewal and proliferation of neoblasts [75]. It will be interesting to find out whether other DDX proteins have direct roles in regulating male germline stem cells in mammals.

Another group of RNA-modulating proteins that are highly expressed in germ cells are Tudor domain “Royal Family” proteins. In *Drosophila*, they are important for germ cell differentiation. Deletion of *Tudor* caused loss of germ cells [76]. In mouse, it was found that Tudor proteins express mostly in meiotic spermatogenic cells (from spermatocytes to elongating spermatids) and often interact with PIWI proteins and participate in the regulation of piRNA biogenesis, retrotransposon repression and DNA methylation. Several Tudor proteins in the germ cells are found to localize in RNA granules and the CB, suggesting their roles in RNA-protein complex formation and mRNA regulation [67]. Mutations of Tudor genes have been shown to cause defects of male germ cell development at early meiotic stages and disruption of TE repression and mRNA stabilities [55, 77]. Despite their wide range of expression during germ cell development and participation in RNA metabolism, it is still not clear whether Tudor proteins directly take part in regulating stem cell functions. Their mode of activities in different cells and context requires further investigation.

## Conclusions and perspectives

RNA-binding proteins possess conserved protein domains that facilitate their interactions with ribonucleic acids [78]. It is estimated that mammalian cells contain over a thousand RBPs with a dozen different RNA-binding domains. However, the number of RBPs and their functional diversity are becoming increasingly complex. New RBPs with canonical RNA-binding motifs as well as novel RNA-binding proteins with no known domains are being discovered [3, 79, 80]. Mutations in many of the RBPs have been linked to human pathologies, including aging and cancer, as well as neurological and muscular disorders [81]. As demonstrated by the RBPs in mouse germ cells, different RBPs can accomplish their functions via different mechanisms. They can either repress or activate protein translation of mRNAs by binding with different proteins that modify the untranslated regions of mRNAs. Through conserved sequence motifs, RBPs often regulate mRNAs with the same sequence features and thus increase the efficiency of regulation by a single protein. How the specificity and functional diversity (repression vs. activation) of RBPs are achieved is one of the central issues regarding molecular mechanisms that govern RBPs.

Although the importance of transcription factors and transcriptional regulation of gene expression are widely accepted, post-transcriptional and translational regulations via RNA-binding proteins have several advantages. First, mRNAs are regulated at multiple steps during their life cycle, providing more opportunities to modulate their functionality with flexibility and specificity for the interpretation of genetic information. As RBPs can participate in every steps of mRNA metabolism, molecular mechanisms that govern their functions can be more versatile. Second, unlike modifications of genome itself which often lead to permanent changes of cells’ gene expression status and aberrant changes can cause inheritable damages, post-transcriptional and translational regulations provide fine tuning of gene expression without changing cellular identity at the genome level. This could be important for intermediate cell types such as transient amplifying stem cells and cells in post-cell cycle states. Third, since mRNAs that already exist in the cells can be subjected to functional modifications, translational regulation can occur in a timely fashion in response to both intrinsic and extrinsic stimuli. In fact, signaling pathways that are activated under different nutritional, energy, and stress status often elicit their effects through regulation of protein translation, such as AMPK and mTORC signaling pathways. Fourth, proteins often function in different subcellular localizations in geometrically asymmetric cells, such as neurons, haploid spermatids, and epithelial cells. These require mRNAs to be modulated in response to localized signals. During development, post-transcriptional and translational regulations offer more subtle control for growth and differentiation of cells. The gradual changes of functionality of a particular cell type will lead to eventual permanent change of cell identity as during the differentiation of stem cells.

Many challenges lie ahead in the study of RBP functions. Current knowledge on RBPs coming from genetic, molecular, biochemical, and bioinformatics research have facilitated our understanding of their physiological functions, protein-protein interactions, and domain-functional annotations. However, many RBPs are multifunctional and dynamically regulated within cells. Systems that allow real-time observation of RBPs at subcellular or single molecule resolution would be required for dissecting the temporal-spatial changes of RBPs under different conditions. For the same reason, animal models that allow analyses of specific functions of RBPs in particular cell types and developmental stages need to be established in order to reveal the precise mechanisms by which they function. Recent development of cutting-edge technologies has added important compliments to explore RBPs’ role on an unprecedented scale. These include genome-wide next-generation sequencing to dissect the exact quantity and composition of RNA species in a cell at particular developmental times [82], RNA interactome-capturing to systematically analyze RBPs to identify the regulators that determine translational status of a cell [3, 79, 80], ribosomal-profiling for dissecting translational status of cellular mRNAs [83], and proteomic analyses to uncover the protein species and changes that occur during self-renewal and differentiation of stem cells [84]. Exciting discoveries are surely to come in years ahead of us.

## Abbreviations

ASC: adult stem cell
CDS: coding sequence
PGC: primordial germ cell
RBP: RNA-binding protein
RNP: ribonucleoprotein
SSC: spermatogonial stem cell
TE: transposable element
UTR: untranslated region
